# Supplementary material for: Implications of Inflammatory and Oxidative Stress Markers in the Attenuation of Nocturnal Blood Pressure Dipping
Source: J Clin Med. 2023 Feb 18;12(4):1643. doi: 10.3390/jcm12041643 (PMC9959900; doi:10.3390/jcm12041643)
Supplement: Supplementary file 1 [file jcm-12-01643-s001.zip › jcm-2146616-supplementary.pdf]

S1 Table. Correlations of 24-h blood pressure indices with inflammatory and redox markers.

Inflammatory markers				Redox markers			
Variables	r	*p*-value	SE	Variables	r	*p*-value	SE
Nocturnal systolic blood pressure (mmHg)							
ESR (mm/h)	0.164	0.011	0.067				
Alpha-1-globulins (g/dL)	0.224	0.027	0.085				
Nocturnal diastolic blood pressure (mmHg)							
ESR (mm/h)	0.145	0.025	0.065				
Alpha-1-globulins (g/dL)	0.174	0.089	0.087				
Nocturnal pulse pressure (mmHg)							
Alpha-1-globulins (g/dL)	0.190	0.063	0.116	Vitamin E (μg/mg TC)	0.178	0.007	0.067
TPS antigen (U/L)	0.206	0.055	0.124				
Beta-2-microglobulin(mg/dL)	0.137	0.035	0.052				
Daytime systolic blood pressure (mmHg)							
Alpha-1-globulins (g/dL)	0.243	0.017	0.107				
Daytime pulse pressure (mmHg)							
Alpha-1-globulins (g/dL)	0.190	0.062	0.137	Vitamin E[a] (μg/mg TC)	0.229	< 0.001	0.059
Nocturnal systolic blood pressure dipping							
ESR[a] (mm/h)	-0.120	0.065	0.066				
Beta-globulins[b] (g/dL)	-0.289	0.004	0.102				
Beta-2-microglobulin[a] (mg/dL)	-0.151	0.007	0.068				
Gamma-globulins[b] (g/dL)	-0.391	< 0.001	0.082				
Nocturnal diastolic blood pressure dipping							
Alfa-2-globulins [b] (g/dL)	0.227	0.025	0.085	Cu[a] (μg/dL)	-0.129	0.048	0.072
TPS antigen[a] (U/L)	-0.195	0.070	0.103				
Gamma-globulins[b] (g/dL)	-0.241	0.017	0.083				

Day to night pulse pressure gradient

Vitamin E[b] (µg/mg TC)	0.111	0.094	0.077
Cu/Zn ratio[a]	-0.127	0.052	0.063
Zn[a] (µg/dL)	0.182	0.005	0.054

[a]Correlation with the percentage difference ((daytime–night-time)/daytime) of each blood pressure index.

[b]Correlation with the absolute difference (daytime–night-time) of each blood pressure index. ESR—Erythrocyte sedimentation rate. TPS—Tissue polypeptide specific. TC—Total cholesterol. Cu—Copper. Zn—Zinc. mmHg—millimeter of mercury. mm—millimeter. h—hour. g—gram. dL—deciliter. U—unit. L—Liter. r—Pearson correlation coefficient. SE—Standard error.
